# Response of the Glutathione (GSH) Antioxidant Defense System to Oxidative Injury in Necrotizing Enterocolitis

**DOI:** 10.3390/antiox12071385

**Published:** 2023-07-05

**Authors:** Alena Golubkova, Tyler Leiva, Katherine Snyder, Camille Schlegel, Sarah M. Bonvicino, Martin-Paul Agbaga, Richard S. Brush, Jason M. Hansen, Peter F. Vitiello, Catherine J. Hunter

**Affiliations:** 1 Division of Pediatric Surgery, Department of Surgery, University of Oklahoma Health Sciences Center, Oklahoma City, OK 73104, USA; 2 Lipid Analysis Core, Department of Ophthalmology, Dean McGee Eye Institute, University of Oklahoma Health Sciences Center, Oklahoma City, OK 73104, USA; sarah-bonvicino@ouhsc.edu (S.M.B.);; 3 Department of Cell Biology and Physiology, Brigham Young University College of Life Sciences, Provo, UT 84602, USA; 4 Section of Neonatal-Perinatal Medicine, University of Oklahoma Health Sciences Center, Oklahoma City, OK 73104, USA

**Keywords:** oxidant agents, oxidative stress, necrotizing enterocolitis, intestinal epithelial injury, glutathione redox potentials, lipid peroxidation, glutathione peroxidase 4, redox signaling

## Abstract

Necrotizing enterocolitis (NEC) is a neonatal intestinal disease associated with oxidative stress. The targets of peroxidation and the role of the innate intestinal epithelial antioxidant defense system are ill-defined. We hypothesized that oxidative stress in NEC correlates with oxidized GSH redox potentials, lipid peroxidation, and a dysfunctional antioxidant system. **Methods**: Intestinal samples from infants +/− NEC were generated into enteroids and incubated with lipopolysaccharide (LPS) and hypoxia to induce experimental NEC. HPLC assayed GSH redox potentials. Lipid peroxidation was measured by flow cytometry. Immunoblotting measured glutathione peroxidase 4 (Gpx4) expression. **Results**: GSH redox potentials were more oxidized in NEC intestinal tissue and enteroids as compared to controls. Lipid radicals in NEC-induced enteroids were significantly increased. Human intestinal tissue with active NEC and treated enteroid cultures revealed decreased levels of Gpx4. **Conclusions**: The ability of neonatal intestine to mitigate radical accumulation plays a role in its capacity to overcome oxidative stress. Accumulation of lipid radicals is confirmed after treatment of enteroids with NEC-triggering stimuli. Decreased Gpx4 diminishes a cell’s ability to effectively neutralize lipid radicals. When lipid peroxidation overwhelms antioxidant machinery, cellular death ensues. Identification of the mechanisms behind GSH-dependent enzyme dysfunction in NEC may provide insights into strategies for reversing radical damage.

## 1. Introduction

Necrotizing enterocolitis (NEC) is a devastating gastrointestinal emergency that affects 4–7% of newborns admitted to the Neonatal Intensive Care Unit (NICU) [1]. NEC preferentially occurs in premature and very low birth weight infants (less than 1.5 kg), although it can also affect full-term patients. Both treatment of the active disease and management of the associated long-term complications (i.e., short bowel syndrome) convey a significant clinical task for the patient, family, and involved medical care providers. As compared to other disorders of prematurity (i.e., bronchopulmonary dysplasia), the morbidity associated with NEC has not shown any major improvements with advances in neonatal care [2]. Additionally, the estimated cost of NEC management in the United States ranges between USD 500 million and USD 1 billion annually [3]. Despite years of advancements and resource allocation, NEC remains responsible for 1 in 10 of all neonatal deaths [4].

Research focused on the underlying pathophysiology of NEC has identified several mechanisms that are involved in disease susceptibility. Introduction of formula feeding and increased caloric density of the latter, subsequent changes to the gastrointestinal microbiome, a leaky and immature intestinal barrier, and a dysregulated inflammatory and immune response to host–pathogen interactions in the gut have all been implicated as inciting factors for NEC. It has also been established that there is a role for oxidative distress in NEC. Previous data has shown that nitrosative stress in NEC promotes intestinal injury, impairs cellular repair mechanisms, and favors apoptosis [5]. Most of these studies have focused on rat and cell line models, and they have demonstrated that a rise in inducible nitric oxide synthase (iNOS) expression is a potential driver for the increased inflammatory response and apoptosis in NEC [6]. 

Unbalanced cellular redox aberrations are associated with disease, including NEC, and it may promote hyperinflammation, impair restitution, and favor cellular death. However, there are also intrinsic defense mechanisms in place to counteract oxidative stress and diminish the buildup of toxic radicals. Within the antioxidant network, the glutathione-dependent (GSH) defense system is of particular interest. GSH is considered one of the essential master antioxidants because of its multiple studied functions in the maintenance of intracellular redox homeostasis, growth and repair promotion, and maturation of tissue function. It is a recyclable molecule, abundant in the body in every organ, including intestinal tissue. GSH-dependent molecules (i.e., glutathione peroxidase family) make up one of the cell’s defense mechanisms against oxidative injury. GSH-dependent enzymes detoxify the cell from buildup of intracellular toxic radicals, utilizing glutathione’s reducing power in the process and converting it from reduced GSH to oxidized glutathione disulfide (GSSG). Earlier studies in rodent NEC models and IEC-6 cells (rat intestinal epithelial cells) have shown that several GSH-dependent antioxidants are upregulated with experimental disease induction, while chemical inhibition of GSH leads to increased cell death [7]. 

Redox tone plays an important role in both inter- and intracellular signaling, promoting repair and growth as opposed to pathologic processes (i.e., ischemia/reperfusion, infection, immune response, cancer, and aging) [8]. In redox homeostasis, a dynamic balance between oxidant production and the antioxidant response is imperative. Redox tone exists on a spectrum between oxidative eustress (physiologic balance of oxidants/antioxidants), which is beneficial for cellular health, and oxidative stress or distress, where pathologic production of oxidant species overwhelms intrinsic antioxidant machinery that should be providing balance and regulation [9,10]. Consequently, the redox environment can shift from a more reduced state to an oxidized state. It has previously been demonstrated that the cellular redox environment is crucial in the regulation of cellular proliferation and maturation, favoring growth in absence of oxidative stress [11]. Intracellular redox tone is measured by comparing the fractions of reduced GSH to oxidized GSSG, from which GSH redox potentials (Eh GSH/GSSG) are derived. More reduced or negative glutathione redox potentials favor cellular proliferation, whereas the opposite is true in a more oxidative state, which can be inferred in the oxidative stress of NEC. A complete understanding of the capacity of the GSH antioxidant system in regulating redox aberrations in human NEC is lacking. A previous clinical study compared total GSH levels in infants affected by NEC to healthy controls by measuring this in blood samples [12]. No significance was found in GSH blood levels among the two groups. Given the primary site of injury in NEC is the intestine, the functional capacity and quantity of GSH likely cannot be evaluated only by looking at total circulating GSH. Current basic science literature remains limited in the characterization of the GSH defense system in its response to NEC. 

Herein, we investigate the role of the intestinal specific GSH defense system. We utilize human intestinal tissue collected from infants with active NEC and primarily derived human enteroid NEC models to investigate the response of the GSH defense system in vivo and under experimental conditions. We hypothesized that NEC-affected tissue and enteroid lines will show evidence of oxidative injury and deregulation of specific GSH-dependent enzymes that are responsible for detoxifying affected cells, as measured by increasingly oxidized GSH redox potentials. This is a novel line of investigation in human tissue and NEC models. We further analyzed whether lipid peroxidation is one of the potential significant targets of oxidative injury. Finally, we quantified the response of glutathione peroxidase 4 (Gpx4), an important GSH-dependent enzyme responsible for neutralizing lipid radicals [13]. We postulated that lipid radical accumulation may occur when cellular defense mechanisms targeted against their accumulation fail to function efficiently and clear the toxic products of lipid peroxidation (i.e., deregulation of Gpx4 in abundance, maturity, or oxidative-injury related modification). This has downstream implications for cell fate and may be a new pathologic pathway to further help describe what leads to overwhelming cell death in terminal NEC pathophysiology.

## 2. Materials and Methods

**Human intestinal sample collection.** Institutional Review Board approval (#11610-11611) was obtained prior to commencement of human tissue collection. After parental consent, a section of pathologic intestinal tissue was collected from infants undergoing clinically indicated bowel resections at the Oklahoma Children’s Hospital (Oklahoma City, OK, USA). Indications for resection included Stage 3 surgical NEC as well as resections for non-inflammatory reasons, such as congenital gastrointestinal malformation (i.e., gastroschisis-related stricture or atresia requiring repair). The latter were used as control tissue for comparison. Gestational age at birth, age at surgery, intestinal section (i.e., ileum), gender, and indications for surgical resection were noted for each sample included. Samples from 27 infants were analyzed in the comparative studies described below, including ten infants with active NEC and 17 infants with non-NEC resections. The majority of infants had a history of prematurity, (gestational age range from 23 to 37 weeks), except for two who were born full term (40 weeks) but required intestinal resections for atresia (part of control group). See Appendix A Table A1 for a comparative summary of basic clinical characteristics for included cases. All intestinal samples were sectioned and snap frozen in liquid nitrogen prior to further processing. 

Enteroid lines were propagated from two of the human ileal specimens, both resected for active surgical NEC. Both lines had history of prematurity, born at 34 and 23 weeks, and underwent resections at 42 and 34 weeks, respectively. Birth weights were 1.79 and 0.55 kg. Both patients passed away at index hospitalization due to total bowel necrosis and respiratory failure, respectively. 

**Harvest and culture of enteroids from human intestinal samples.** Tissue collected for enteroid line derivation was immediately washed in cold Dulbecco’s phosphate-buffered saline (DPBS, Sigma Life Science, #D1408, St. Louis, MO, USA) and processed on the same day as collection. Stem cell crypts were isolated for enteroid culture generation using a previously described protocol for enteroid processing [14]. Enteroid cultures were suspended in basement membrane matrix or Matrigel (Corning, #CB-40230C, Corning, NY, USA) domes and grown in 50% L-WRN conditioned media prepared in our lab, supplemented with 50 ng/mL epidermal growth factor (Millipore Sigma, #GF144, Burlington, MA, USA), 1 mM N-Acetylcysteine (Millipore Sigma, #A9165-5G, Burlington, MA, USA), 500 nM A-83-01 (R&D Systems, #2939/10, Minneapolis, MN, USA), 10 μM SB202190 (Millipore Sigma, #S7067-5 MG, Burlington, MA, USA), 10 mM Nicotinamide (Millipore Sigma, #N0636-100G, Burlington, MA, USA), and 10 nM [leu] 15-gastrin 1 (Millipore Sigma, #G9145-.1 MG, USA). Enteroids were passaged weekly and experimented on once they reached maturity. All studies were completed between passages 5–10 for all described experiments in this study.

**NEC induction in human enteroids.** Enteroid cultures were grown in Matrigel domes (Corning, #CB-40230C, Corning, NY, USA), as previously described, until maturity (around seven days from passage). For experimental NEC induction, enteroids were subjected to treatment with 100 ug/mL lipopolysaccharide added to the media and hypoxic conditions (1% Oxygen) provided by the modular incubator chamber from Billings-Rothenberg, Inc. (Del Mar, CA, USA). N-Acetylcysteine was not supplemented into experimental media for either the control or the treated cultures during experimentation, given its proposed benefits against oxidative injury. Treatment duration lasted from 24 to 48 h. Control enteroids were harvested at both commencement (0 h) and end of experiment (48 h) to follow baseline redox changes within the cultures. Each sample was considered to be three pooled domes of enteroids, with at least three biological replicates per treatment group. 

**HPLC analysis for measurement of GSH redox potentials and protein bound GSH in human tissue and enteroid cultures.** Methodology for HPLC analysis was previously described and modified by Harris and Hansen [15]. Snap frozen intestinal tissue and enteroid pellets harvested for GSH redox potential measurements were preserved in cold 5% perchloric acid buffer containing 10 μM γ-glutamyl-glutamate at −80 °C until processing. Samples were solubilized in 100 mM sodium hydroxide, and protein concentrations were quantified using BCA assay. GSH and GSSG concentrations were determined using reverse-phase high performance liquid chromatography (HPLC (Waters 2695 Alliance Separations Module with a Supercoil LC-NH_2_ column). Peak detection was made using a Waters 2474 fluorescence detector (335 nm/518 nm). Molarity values of GSH and GSSG were determined through standardization with the internal standard (γ-glutamyl-glutamate) and sample protein concentrations. Redox potentials were derived using the Nernst equation. 

For protein bound GSH, *S*-glutathionylated proteins (PrSSG) pellets collected from samples during collection were washed with 5% perchloric acid and then re-pelleted, and the supernatants were removed. Pellets were treated with 0.5 mL 0.1 M NaOH to neutralize the perchloric acid, and the pellets were resuspended. An aliquot (0.25 mL) was then added to an equal volume of 0.1 M phosphate buffer (pH 6.0) containing 5 mM dithiothreitol, and the solution (final pH approximately 7.6) was incubated at room temperature for 30 min. Following the incubation, 0.5 mL of 10% perchloric acid/boric acid containing the internal standard was added. Samples were then processed and derivatized as described above via HPLC. Data is presented as GSH nmol/mg protein.

**Lipid peroxidation measurements via flow cytometry in human enteroid NEC model.** Enteroid cultures were subjected to our previously described experimental NEC conditions for 48 h. Matrigel domes were then dissolved and the enteroid cultures were suspended in growth media with the Image-It^®^ Lipid Peroxidation Sensor (#C10445, Life Technologies, Carlsbad, CA, USA) at a manufacturer-recommended concentration of 10 µM. The Lipid Peroxidation Sensor is a fluorescent reporter based on BODIPY^®^ 581/591 C11 reagent which, in live cells, shifts from red to green with increasing lipid peroxidation products, providing a ratiometric indication of lipid radical accumulation. Enteroids were incubated at 37 degrees Celsius for 30 min with the sensor, after which time the cultures were washed in 1× phosphate buffered saline (PBS) and further quickly processed into single cells using trypsin (ThermoFisher Scientific, #78840, Rockford, IL, USA) as the dissociation agent. Cells were analyzed with flow cytometry (Stratedigm S1200Ex flow cytometer platform, Stratedigm-3) at a total of 1000 events collected per sample, with a 488 nm laser excitation and fluorescence emission collected from 530/30 nm (green) and 488 nm laser excitation and fluorescence emission collected from 580/30 nm (red). Ratios of red/green fluorescence intensities were quantified and compared among control and NEC-induced enteroid groups (each run in triplicate). A decrease in red/green fluorescence intensity ratios corresponded with an increase in lipid peroxidation in the sample. A positive control was included, using enteroid cultures treated with 100 µM cumene hydroperoxide for 2 h prior to analysis.

**Lipid profiling of fatty acid composition in intestinal tissue samples.** Total lipid extraction was performed using a protocol described by Folch et al., in which samples were homogenized in methanol [16]. Fifty nanomoles of pentadecanoic (15:0) and heptadecanoic (17:0) acids were added as internal standards. Lipid extracts underwent acid hydrolysis/methanolysis by heating at 100 degrees Celsius in 16% *v*/*v* concentrated hydrochloric acid (HCl) in methanol to generate fatty acid methyl esters (FAMEs). FAMEs were extracted in hexane and purified via thin-layer chromatography (TLC) based on previously described protocol [17]. FAMEs were identified using an Agilent Technologies 7890A gas chromatograph with a 5975C inert XL mass spectrometer detector (Agilent Technologies, Lexington, MA, USA) [18]. FAMEs were quantified using an Agilent Technologies 6890N gas chromatograph with flame ionization detector [18]. Sample concentrations were determined by comparison to internal standards (15:0 and 17:0). Data are represented as relative mole percent of each fatty acid.

**SDS-PAGE and immunoblotting of protein extracted from intestinal tissue and treated enteroid cultures.** Protein extraction was completed using a RIPA lysis cocktail (1×, Cell Signaling Technology, #9803, Danvers, MA, USA) with phosphatase and proteinase inhibitors (Phosphatase Inhibitor Cocktail 2, Sigma Aldrich, #P5726, St. Louis, MO, USA; Protease Inhibitor Cocktail, Sigma Aldrich, #P8340, St. Louis, MO, USA). Total protein concentrations were quantified using the Pierce^TM^ BCA Protein Assay kit (ThermoFisher Scientific, #23225 and 23227, Rockford, IL, USA). Gels with 10% hand-cast acrylamide (BioRad, #1610156, Hercules, CA, USA) were used for SDS-PAGE. Protein was loaded at 10 μg per well for both proteins derived from intestinal tissue and enteroid NEC studies. Gels were transferred on to nitrocellulose membranes and blocked in 5% milk. Membranes were incubated with primary antibodies overnight at 4 degrees Celsius and for 1–2 h at room temperature with secondary HRP-linked antibodies. Washes were completed with 1× tris-buffered saline, 0.1% Tween^®^ 20 (TBS-T). The primary antibodies used were glutathione peroxidase 4 (Gpx4) at a dilution of 1:5000 (ab125066, abcam, Boston, MA, USA), 4-HNE at a dilution of 1:1000 (MA5-27570, Invitrogen, Danvers, MA, USA), and 4-HHE at a dilution of 1:5000 (MA5-27556, Invitrogen, Danvers, MA, USA). Β-actin served as a reference protein for normalization at a dilution of 1:1000 (#3700, Cell Signaling, Danvers, MA, USA). Secondary antibodies included horse anti-mouse HRP-linked antibody at 1:1000 dilution (#7076s, Cell Signaling, Danvers, MA, USA) and goat anti-rabbit HRP-linked antibody at 1:10,000 dilution (#31460, Invitrogen, Danvers, MA, USA).

**Statistical analysis.** Results are represented as means with SEM as error bars, unless otherwise reported. Statistical analysis and figures were completed using GraphPad Prism 9.5.0 software. Comparative analyses were run using Student’s *t*-test or analysis of variance (ANOVA), as appropriate. All compared groups had at least three biological replicates and were run in triplicate to improve rigor of results.

## 3. Results

### 3.1. GSH/GSSG Redox Potentials in Human Intestinal Samples

**Figure 1:**
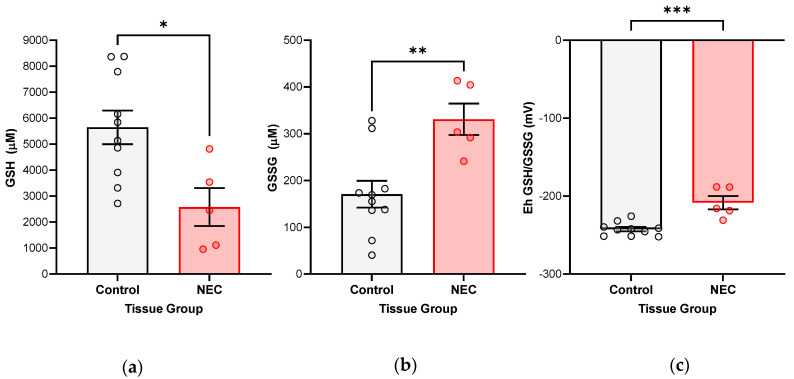
**GSH redox potentials compared between small bowel specimens from infants with active NEC (N = 5) and tissue collected for other, surgically indicated reasons (Control, N = 10), without history of NEC.** The tissue was snap frozen at the time of harvest and preserved in perchloric acid buffer containing γ-glutamyl-glutamate. Samples were analyzed by high-performance liquid chromatography (HPLC). Intracellular GSH and GSSG concentrations were determined, from which redox potentials were further derived: (**a**) Intestinal samples with active NEC show significantly decreased reduced intracellular GSH fractions (* *p*-value 0.0122); (**b**) significantly increased oxidized intracellular GSSG fractions (** *p*-value 0.0048); (**c**) and, consequently, more oxidized GSH redox potentials (*** *p*-value 0.0003). Groups were compared using Student’s *t*-test statistical analysis (*p*-value < 0.05 considered significant).

Intestinal samples from infants with active NEC show evidence of oxidative stress as confirmed by the significantly oxidized GSH redox potentials from our HPLC analysis (Figure 1). Furthermore, reduced GSH concentrations are significantly decreased while oxidized GSSG is significantly increased when comparing NEC tissue to control tissue. 

### 3.2. GSH/GSSG Redox Potentials in a Human Enteroid NEC Model

**Figure 2:**
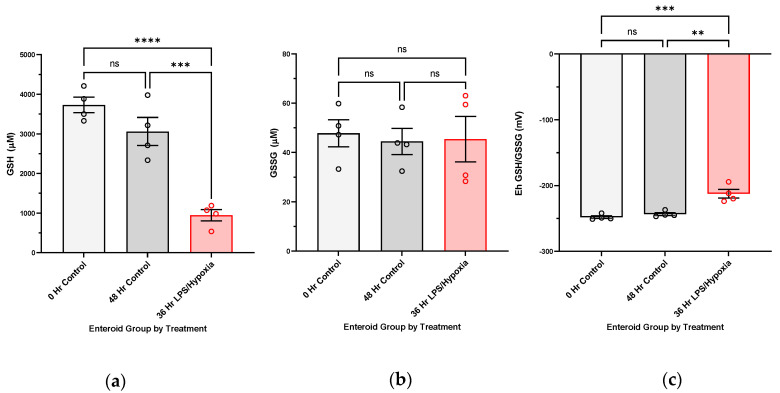
**GSH redox potentials in human enteroid cultures after experimental NEC induction (36 h LPS/Hypoxia, N = 4), compared to control enteroid cultures at the beginning and end of study (0 h Control, N = 4 and 48 h Control, N = 4).** Enteroid cultures were propagated from an infant intestinal specimen resected for active NEC, exposed to NEC-inducing stimuli at maturity over a 0–48-h period, and, finally, pellets were collected and preserved in perchloric acid buffer containing γ-glutamyl-glutamate. Analyses was completed with high-performance liquid chromatography (HPLC). Intracellular GSH and GSSG concentrations were determined, from which redox potentials were further derived: (**a**) Enteroids show significantly decreased reduced intracellular GSH at 36 h of exposure to NEC stimuli (**** *p*-value < 0.0001, *** *p*-value 0.0005), while control enteroids remain with similar reduced GSH fractions from 0 to 48 h of incubation; (**b**) no significant increase in oxidized intracellular GSSG can be followed; (**c**) enteroids show significantly oxidized GSH redox potentials (*** *p*-value 0.0005, ** *p*-value 0.0013) at 36 h of NEC-induction. GSH redox potentials do not show changes in redox state from 0 to 48 h in the control cultures. Groups were compared using ANOVA (*p*-value < 0.05 considered significant). See Appendix A Figure A1 for complete results of redox potentials collected (0, 24, 36, 48 h).

Exposure of enteroid cultures to NEC-inducing experimental conditions (LPS and hypoxia) leads to increasingly oxidized GSH redox potentials, mirroring the relationship shown in tissue with active NEC versus control. Interestingly, Figure 2 shows that the changes in GSH redox potentials are driven by a decrease in GSH, as opposed to a rise in oxidized GSSG. Control cultures at 0 and 48 h of the experiment have comparable GSH redox potentials.

### 3.3. Protein Glutathionylation (Pr-SSG) in Human NEC Tissue and NEC-Induced Enteroids

**Figure 3:**
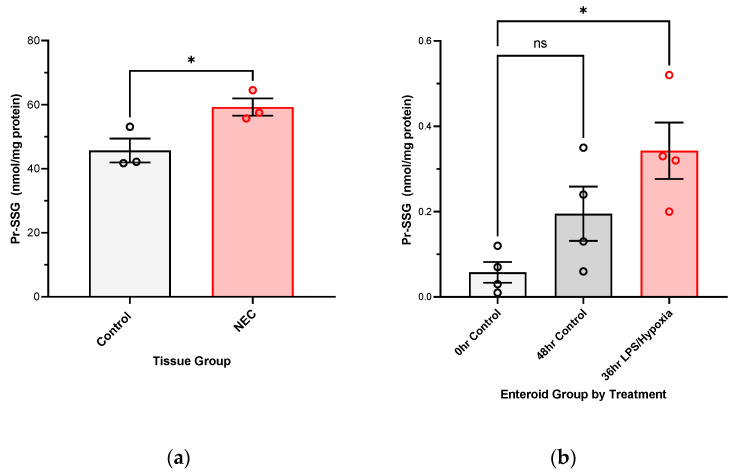
**Protein glutathionylation is evident in** (**a**) primary intestinal tissue samples resected for surgical NEC (* *p*-value 0.0417, Control N = 3, NEC N = 3), as well as (**b**) human enteroid cultures after 36 h of in vitro NEC induction (* *p*-value 0.0119, N = 4 separately grown and treated, pooled enteroid culture samples per group. Each enteroid culture sample is a pooled sample of 3 enteroid domes that includes approximately 150 enteroids grown to maturity in a three-dimensional matrix dome). Analysis carried out with Student’s *t*-test and ANOVA, respectively, with *p*-value < 0.05 considered significant.

Both intestinal samples and primary human enteroid cultures show evidence of protein glutathionylation if resected for active NEC or exposed to in vitro NEC stimuli, as compared to their respective control groups, as shown in Figure 3. 

### 3.4. Lipid Peroxidation Is a Result of Experimental NEC Induction in Human Enteroid Cultures

**Figure 4:**
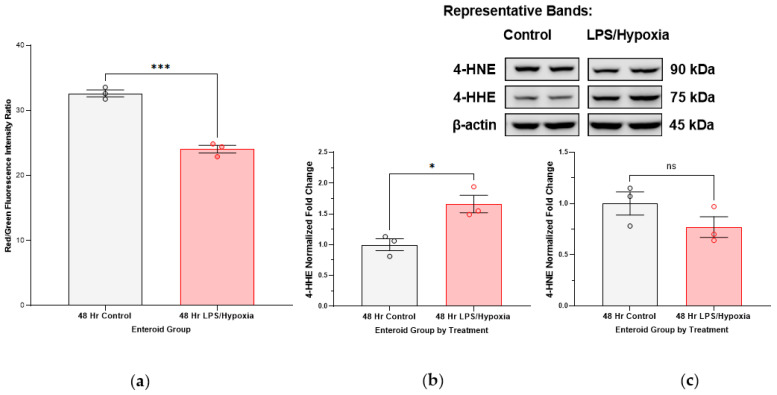
**Measures of lipid peroxidation in a human NEC enteroid model:** (**a**) Enteroids propagated from human intestinal tissue with surgical NEC were treated for 48 h with LPS and hypoxia to induce in vitro NEC. Both control and treated cultures were harvested at 48 h. Live cell flow cytometry analysis of the cultures after application of a fluorescent lipid peroxidation sensor shows decreased red/green fluorescence ratios, indicative of accumulation of lipid peroxidation products with experimental NEC (*** *p*-value 0.0004). (**b**) 4-HHE protein expression analyzed by SDS-PAGE and immunoblotting of protein extracted from NEC-treated enteroids increases with time after exposure to NEC-stimuli, most prominent at 48 h of incubation (* *p*-value 0.0114). The 75 kDa band is presented here, as expected per antibody manufacturer, but another 20 kDa band can also be followed, potentially representing an adduct formation (without significant change in expression). (**c**) 4-HNE protein expression in this enteroid culture did not change with treatment time. Each representative group includes N = 3 of separately grown and treated enteroid culture samples. Each enteroid culture sample is a pooled sample of three enteroid domes that includes approximately 150 enteroids grown to maturity in a three-dimensional matrix. Analysis carried out with Student’s *t*-test and ANOVA, as appropriate, with *p*-value < 0.05 significant.

After 48 h of exposure to LPS and hypoxia, enteroid cells show lower red/green fluorescence intensity ratios as measured by flow cytometry, showing that there is a significant accumulation of lipid peroxidation products (Figure 4a). Furthermore, lipid peroxidation is associated with the formation of toxic lipid aldehydes such as 4-HNE (4-hydroxy-2-nonenal, which is a result of peroxidation of n-6 PUFAs) and 4-HHE (4-hydroxy-2-hexenal, a result of peroxidation of n-3 PUFAs). Protein analysis via Western blotting on our enteroid cultures shows that there is an uptrend in expression of 4-HHE in longer treated enteroids as compared to controls. No significant trend was noted in the expression of 4-HNE in this experiment (Figure 4b,c).

### 3.5. Lipid Profiling of Human Intestinal Tissue with Active NEC versus Control Diagnoses

**Figure A2:**
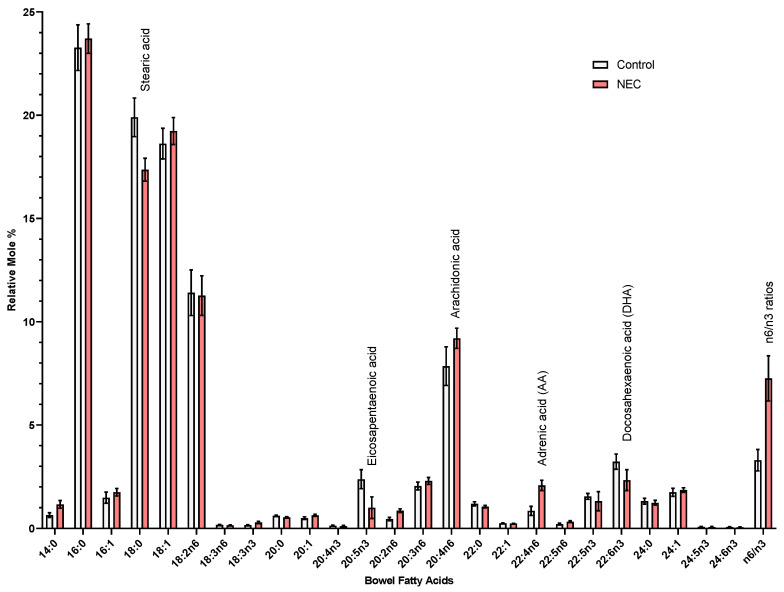
**Total bowel fatty acid composition of intestinal tissue with NEC (N = 8) vs. control diagnoses (N = 6)**. All included resections came from infants born prior to 40 weeks gestation. Control diagnoses included resections completed for jejunal atresia and gastroschisis/dysfunctional bowel, with four of the subjects never exposed to enteral nutrition prior to surgery. All NEC tissue came from premature infants, with diagnosis of NEC at 30–35 weeks corrected gestational age, all on fortified breastmilk or formula 1–3 days prior to developing symptoms.

Fatty acid composition analysis of human intestinal samples with active NEC versus control diagnoses shows trends in several PUFAs. See Appendix A Figure A2 for results. Overall, there is a trend towards increased omega-6 (n6) to omega-3 (n3) ratios in intestinal samples with active NEC history. NEC samples also show a trend towards increased expression of omega-6 fatty acids such as arachidonic acid and adrenic acid, as well as lower expression of omega-3 fatty acids such as eicosapentaenoic acid and docosahexaenoic acid (DHA). Of note, the origin tissue of the enteroid line that was propagated for the experiments described in this study was also analyzed, showing an increased proportion of DHA, which correlates with the increased 4-HHE expression in protein analysis described in Figure 4b, as 4-HHE is a product of omega-3 PUFA peroxidation. Furthermore, it is suggestive that the lipid profile is retained in the early passages of enteroid cultures to resemble that of the host tissue it was propagated from.

### 3.6. Gpx4 Expression in Human Intestinal Samples and NEC Enteroid Model

**Figure 5:**
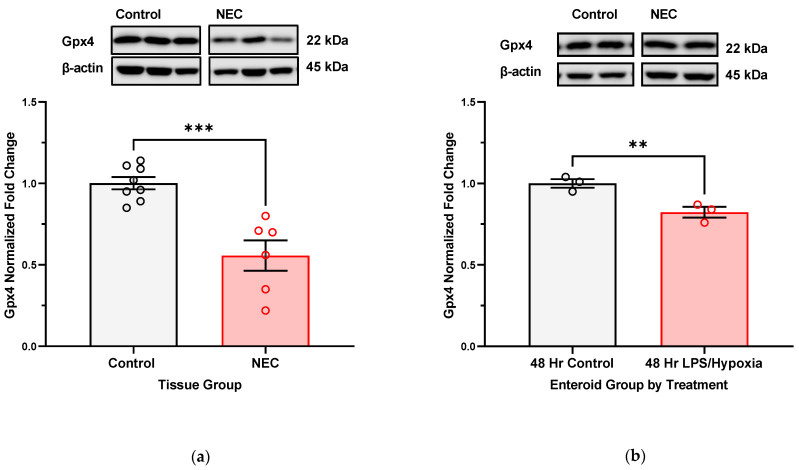
**Comparing Gpx4 protein expression in human intestinal samples and enteroid cultures, NEC versus control**. Protein extracted from intestinal samples with active NEC (N = 5) and specimens resected for other surgically indicated reason (N = 8) underwent SDS-PAGE and immunoblotting for Gpx4 expression analysis. Enteroid cultures propagated from intestine with active NEC were also subjected to in vitro NEC stimuli for 48 h, after which protein was extracted and also underwent protein analysis (3 wells of cultures combined per sample, with N = 3 per comparison group). Gels run quadruplicate for each study. (**a**) Gpx4 expression is decreased in samples affected by NEC (*** *p*-value 0.0004); (**b**) Gpx4 expression is also decreased in enteroid cultures 48 h after NEC induction in vitro (** *p*-value 0.0027). Comparison carried out using Student’s *t*-test, *p*-value < 0.05 being significant.

Intestinal tissue collected at the time of active NEC shows significantly lower protein expression of glutathione peroxidase 4 (Gpx4), a GSH-dependent selenoprotein responsible for converting toxic lipid radicals to neutral alcohols (Figure 5a). A significant drop in expression can also be appreciated in enteroid cultures that have been subjected to NEC-inducing stimuli for up to 48 h (Figure 5b).

## 4. Discussion

The role of oxidative injury in the pathogenesis of NEC has been proposed in the literature but not fully explained. Neither has the response of the innate glutathione antioxidant defense system been comprehensively characterized. It is established, though, that oxidative stress is partly responsible for cellular injury that is seen in NEC and other inflammatory bowel disease such as Crohn’s and experimental DSS-colitis [19,20,21,22]. Aberrations in the redox state of a cell are also one of the main regulatory checkpoints that may usher a cell towards death as opposed to repair and restitution [7,11,22,23]. Studies of human intestinal tissue have yet been able to confirm glutathione-dependent antioxidant system dysfunction in NEC, including changes in the GSH-controlled redox environment (i.e., GSH redox potentials). Furthermore, measures of oxidative stress in NEC-affected infants are mostly completed by blood analyses [12,20]. In this investigation, we aimed to investigate the role and function of the glutathione antioxidant defense as well as the potential targets of oxidative injury in the intestine, the target tissue affected by NEC. 

We have shown, for the first time, that NEC injury of the intestine is associated with increasingly oxidized GSH redox potentials in the target intestinal tissue in vivo. We have also been able to confirm this in an experimental human NEC enteroid model. A shift towards increasingly oxidized GSH redox potentials in both tissue and enteroids demonstrates that glutathione-dependent changes are occurring intracellularly in response to the disease. The implication of an oxidized redox intracellular environment is inhibition of cellular proliferation and increased regulatory cell death [7,11]. Consequently, the ability of NEC-affected intestinal tissue to repair itself may be impaired, leading to eventual necrosis and perforation as seen in the more severe, surgical NEC stages. In addition, with the derivation of protein glutathionylation, we have showed that both human intestinal tissues affected by NEC and experimental NEC enteroid model have increasing levels of glutathionylated protein. S-glutathionylation can lead to modification of target enzymes [24]. 

Furthermore, we show that lipids are targets of oxidative injury in NEC. Polyunsaturated fatty acids (PUFAs) are abundant in the cell (e.g., lipid bilayer of the cell membrane as well as fatty acids part of inflammatory signaling cascades) and have been implicated as main targets/substrates for lipid peroxidation under oxidative stress in studies of other intestinal disease models [25]. Lipid radicals that form as a result of this peroxidation can be downstream signaling molecules for cell demise (i.e., oxidized arachidonic and adrenic phosphatidylethanolamines (PEs) in ferroptosis, an iron-dependent form of regulatory cell death that is triggered by accumulation of lipid radicals), as well as have the ability to form protein adducts and modify protein function inside the cell, with eventual cytotoxic consequences (i.e., 4-HNE and 4-HHE) [26,27,28]. Downstream formation of toxic lipid aldehydes, 4-HHE (4-hydroxy-2-hexenal) and 4-HNE (4-hydroxy-2-nonenal) stems from peroxidation products of n-3 and n-6 PUFAs, respectively [29]. Interestingly, we have noted an uptrend in 4-HHE in our immunoblotting more so than 4-HNE. Furthermore, blotting for 4-HNE in active NEC intestinal specimens actually resulted in multiple bands on the membrane (Appendix A). Identification of these bands can help us determine if these multiple bands are a result of 4-HNE adducts and what proteins are affected by these modifications. Theoretically, given 4-HNE is a result of peroxidation of n6 PUFAs, while 4-HHE is a result of n3 PUFA peroxidation, we can likely augment the levels of these lipid aldehydes by changing the baseline supply of PUFAs. Dietary modification/supplementation of PUFAs (i.e., omega-3 docosahexaenoic acid (DHA), omega-6 arachidonic acid) may have a role in the tendency for production of one toxic lipid aldehyde over the other in the face of oxidative stress, depending on which fatty acids have been incorporated into the lipid profiles of the cells [30]. Additionally, when comparing oxidation products of PUFAs in infant formula versus human breastmilk, formula was found to have higher levels of 4-HHE/n-3 PUFA ratios than breastmilk [31]. Levels of malondialdehyde (MDA, another lipid peroxidation marker) were also elevated. Further investigation into lipid profiling of formulas and fortification mixes will help further elucidate if the potential source of oxidant substrates stems from nutritional sources. Supplementation of readily oxidizable PUFAs may exacerbate intestinal inflammation that is seen in NEC. As a comparison to other intestinal disorders, inflammatory bowel disease such as Crohn’s has also been recently tied with westernization of diets. Western diets are rich in inflammatory PUFAs such as omega-6 arachidonic acid. Mayr et al. have shown that intestinal epithelial cells with Crohn’s have Gpx4 dysfunction and signs of lipid peroxidation [32]. Furthermore, the group has been able to trigger Crohn’s-like inflammation similar to the human phenotype in mice that lack one allele of Gpx4 (murine double knockouts are lethal).

Nutritional supplementation of key antioxidants (i.e., anti-inflammatory omega 3 fatty acids, Vitamin E, n-acetylcysteine) can, on the contrary, promote adequate antioxidant defense responses. Breastfeeding with human milk is an example of a nutritional strategy that has been shown to promote a healthy redox environment [10]. HMOs (human milk oligosaccharides) have been extensively studied for their benefits to gut health, promoting a healthy microbiome balance and controlling pathogens, increasing cellular proliferation, modulating inflammatory responses, and with indirect antioxidant function [33,34,35,36]. Targeted supplementation with GSH, for example, as the principal antioxidant for premature infants with antioxidant deficiencies, is being investigated in literature for its therapeutic and preventative potential. Mode of supplementation is also imperative as GSH addition to nutritional modalities such as parenteral nutrition or infant formula has the potential for oxidation of the antioxidant leading to decreased availability [37]. 

With analysis of Gpx4 expression in NEC-affected human intestinal tissue and in NEC-induced enteroids, we show that with longer exposure to NEC-stimuli, there is likely a more significant deregulation of the innate antioxidant defense mechanisms. In the specific case of Gpx4, we see a downregulation in protein expression of this enzyme. Uniquely, it is one of the only GSH-dependent glutathione peroxidases capable of neutralizing lipid radicals into benign alcohols [38]. Consequently, we can infer that in the face of oxidative stress and lipid radical accumulation, one of the main detoxifying enzymes becomes deregulated or dysfunctional. This may be due to decreased production of Gpx4 but can also be a result of degradation or modification (i.e., protein glutathionylation or adduct formation with a toxic lipid aldehyde product such as 4-HHE or 4-HNE). Further specialized characterization of targets of protein glutathionylation and or adduct formation will help identify if Gpx4 is affected by either of these modifications. 

While our study focuses on the consequences of intestinal oxidative injury of NEC, the disease is a multisystemic disorder, with the redox aberrations that start in the intestine but that have the potential to spread and lead to ultimate systemic septic shock and multiorgan failure. The previously mentioned clinical study that measured total GSH in blood samples of infants with and without NEC did not show a significant drop in the antioxidant between healthy infants and those affected by NEC, but it did show a trend towards lower GSH levels with more extensive disease [12]. This prompts further needed research into the temporal relationship of accumulating oxidative distress and cellular signaling and defense response in NEC-affected infants. Deregulation of the antioxidant response is likely a later pathologic dysfunction that occurs as the redox tone shifts towards a more oxidized environment. Furthermore, Bindi et al. have showed that oxidative injury of NEC leads to mitochondrial dysfunction in the liver in a murine model [39]. After experimental induction of NEC with hypoxia, hyperosmolar formula, and LPS administration, mouse pups in the experimental NEC group were compared to breastfed control pups. Interestingly, NEC pups demonstrated depletion of viable and functional liver mitochondria and increased levels of NRF2 in the liver (nuclear factor erythroid 2-related factor 2), a marker of corollary oxidative stress in this study. NRF2 is upregulated in the face of oxidative stress to induce expression of an array of antioxidant genes for downstream function of the antioxidant defense system [40]. The Bindi study group suggests that mitochondrial damage is a key pathologic response to NEC injury on a systemic level. Investigation of mitochondrial response is imperative in the intestine as well, as function of mitochondria is closely tied to cell death pathways and disruption in energy production within affected cells. The increased metabolic requirements of fighting an active infection and hyperinflammation are likely compromised by the mitochondrial damage that occurs with NEC, ultimately leading to systemic shock pathophysiology where the energy demands of the tissue are unable to be met by compromised energy production. This is especially true for preterm infants who already have dysfunctional or immature mitochondria, predisposing them to prematurity-related disorders, such as bronchopulmonary dysplasia [41].

Lipid radical accumulation within a cell and dysfunctional Gpx4 are two of the three inciting criteria that can prompt a cell towards undergoing a relatively newly described form of cell death, ferroptosis [42]. Ferroptosis is an iron-dependent form of programmed cell death that is triggered by three hallmark events, with lipid peroxidation and overwhelming of intracellular antioxidant defense machinery (downregulation of Gpx4) being two of them [43]. The third factor is accumulation of iron within the affected cell, which triggers auto-oxidative injury through catalysis of Fenton chemistry [44]. Free ferrous iron has the potential to form extremely reactive hydroxyl radicals in the presence of hydrogen peroxide. Hydroxyl radicals are nonspecific and have the potential to react with biomolecules such as PUFAs, amplifying the redox aberrations that follow lipid peroxidation, as formed lipid radicals go on to participate in autooxidation [9]. Our future investigation will continue characterizing the role of the glutathione-dependent defense system in NEC, the implications of redox imbalance on cellular signaling with increasing oxidative distress in the disease, the effects of lipid peroxidation on the intracellular redox environment (as well as the potential cytotoxic protein modifications that are possible through lipid aldehyde adduct formation and glutathionylation), modulation of lipid peroxidation byproducts through changes in dietary PUFA supplementation, and the role for ferroptosis as another cell death pathway to describe the terminal pathophysiology associated with necrotizing enterocolitis.

The results of our study are unique in that they are based on analysis of human infant tissue and human-derived enteroid cultures. This provides representative samples that are closest to the patient groups that are most susceptible to NEC. The intestinal samples we worked with were obtained from infants undergoing clinically indicated bowel resections for either active NEC or other gastrointestinal conditions requiring surgical correction. Although these samples provide an opportunity for us to study NEC in the most appropriate experimental setting, there are limitations to this as well. Surgical correction is relatively rare and seldom indicated in the newborn. The amount of tissue available is limited. Basic clinical characteristics (i.e., age, prematurity, clinical conditions) depend on the unique case of a patient requiring surgery, and, thus, while NEC resections may come from patients with relatively similar histories, comparative control samples come from cases that also have surgical pathology, but which do not have to be tied to certain characteristics such as prematurity or birth weight. This results in subject populations that can be relatively similar but not fully matched in all their clinical histories. Additionally, working with fragile NEC-affected tissue poses its own challenges given that the necrotic bowel has altered genetic/protein profiles affected by pathologic degradation and may be difficult to propagate when deriving enteroid cultures. To address these concerns, we worked to include all available control tissue (i.e., increase number of controls to case samples) as a comparison when the experiment required an adequate comparison. 

## 5. Conclusions

Among the proposed mechanisms, oxidative injury and its effects on cellular function have been implicated in NEC but not completely defined. The aim of our study was to further investigate the function of the glutathione-dependent antioxidant system in intestinal tissue affected by this disease. With this set of experiments, we have confirmed that the oxidative stress of NEC has an impact on the function of the glutathione antioxidant system in the intestine, prompting further investigation into the implications this has on the system’s capacity to defend affected tissue from buildup of toxic radicals and the role this has in the later pathophysiology we see in NEC. Deregulation of Gpx4 is impairing the cell’s ability to neutralize the buildup of intracellular lipid radicals in the face of ongoing oxidative stress. Lipid peroxidation is known to preferentially target polyunsaturated fatty acids. Furthermore, toxic derivatives of fatty acid oxidation can lead to adduct formation and modification of key proteins inside the cell, turning off their function or marking them for degradation. Finally, unchecked lipid radical accumulation and gpx4 deregulation are two of the key events that are known to usher an affected cell towards ferroptosis, a relatively new form of iron-dependent regulated cell death. The role of ferroptosis in terminal pathophysiology seen in NEC has not been shown before. Our future investigation will help link how the overwhelming oxidative injury of NEC leads to deregulation of the glutathione defense system, thereby favoring cellular death and impairing repair and proliferation of NEC-affected tissue.

## Figures and Tables

**Figure A1:**
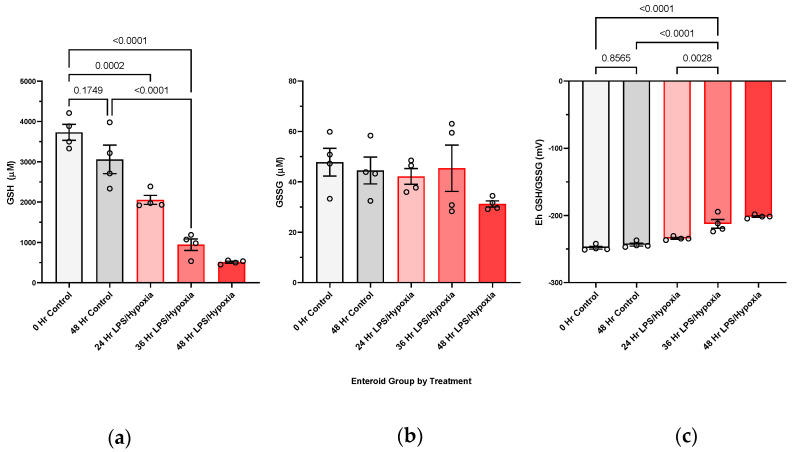
**Expanded results for GSH redox potentials in human enteroid cultures after experimental NEC induction (N = 4, for each collection time point at 24, 36, and 48 h), compared to control enteroid cultures at the beginning and end of study (0 h Control, N = 4 and 48 h Control, N = 4)**. Accompaniment to Figure 1 in main text. Enteroid cultures were propagated from an infant intestinal specimen resected for active NEC, exposed to NEC-inducing stimuli at maturity over a 0–48 h period, and, finally, pellets collected from three domes into one sample and preserved in perchloric acid buffer containing γ-glutamyl-glutamate. Analysis was completed with high-performance liquid chromatography (HPLC). Intracellular GSH and GSSG concentrations were determined, from which redox potentials were further derived: (**a**) enteroids show a stepwise decrease in reduced intracellular GSH, becoming significant at 24–36 h of exposure to NEC stimuli. Untreated, control enteroids remain with similar reduced GSH fractions from 0 to 48 h of incubation; (**b**) no significant increase in oxidized intracellular GSSG can be followed; (**c**) enteroids show significantly oxidized GSH redox potentials at 36 h of NEC induction. GSH redox potentials do not show changes in redox state from 0 to 48 h in the control cultures. Groups were compared using ANOVA (*p*-value < 0.05 considered significant, annotated on the graphs).

## Data Availability

The data presented in this study are available on request from the corresponding author.

## Appendix A

**Table A1:** Summary of basic characteristics for patients from which intestinal samples were included in enteroid and tissue analysis. Numerical characteristics were compared using a Student’s *t*-test, while categorical variables were compared using a Fisher’s exact analysis. *p*-value < 0.05 is considered significant.

	Control (N = 17)	NEC (N = 10)	*p*-Value
Gestational Age at Birth (mean weeks, range)	32.3 (26–40)	28.4 (23–34)	0.033
Birth Weight (mean kg, range)	2.00 (0.60–3.33)	1.14 (0.51–1.79)	0.008
Gender (% female)	41.2%	10%	0.190
Mortality (%)	-	30% ^1^	0.041

^1^ Of three patients who passed in the NEC group, two patients had NEC totalis (defined as complete to near total bowel necrosis with mortality reaching almost 100% in affected patients), and one patient passed from respiratory failure.
